# Primary prevention of cardiovascular disease: The past, present, and future of blood pressure- and cholesterol-lowering treatments

**DOI:** 10.1371/journal.pmed.1002539

**Published:** 2018-03-20

**Authors:** Maarten J. G. Leening, M. Arfan Ikram

**Affiliations:** 1 Department of Epidemiology, Erasmus MC – University Medical Center Rotterdam, Rotterdam, the Netherlands; 2 Department of Cardiology, Erasmus MC – University Medical Center Rotterdam, Rotterdam, the Netherlands; 3 Department of Epidemiology, Harvard T.H. Chan School of Public Health, Boston, Massachusetts, United States of America

## Abstract

In a Perspective, M. Afran Ikram and Maarten Leening discuss the evolving approaches to determining cardiovascular risk.

Shortly after World War II, coronary heart disease was recognized as an epidemic. Cardiovascular disease (CVD) had become the leading cause of death in Western societies. This led to the formation of the National Heart, Lung, and Blood Institute in the United States and initiation of the seminal Framingham Heart Study in 1948. This population-based cohort study on the etiology and consequences of CVD has shed light on many of the well-known causes of CVD. In one of their initial publications in 1961, the investigators from the Framingham Heart Study introduced the concept of “factors of risk in the development of coronary heart disease”, nowadays known as traditional cardiovascular risk factors [1].

The identification of high blood pressure and cholesterol levels as causes of CVD led to the idea of screening and treatment thereof in otherwise healthy persons, in order to halt atherosclerosis and forestall the occurrence of cardiovascular events. Initial treatment recommendations in the 1970s and 1980s were based on the levels of the specific risk factors, with antihypertensive treatment recommended for “virtually all persons with a diastolic blood pressure exceeding 105 mmHg” [2]. However, a gradual diversification then took place in preventive cardiology: hypertension guidelines remained focused on blood pressure levels, whereas cholesterol treatment guidelines moved towards more sophisticated approaches by recommending pharmacological interventions informed by the individual’s cardiovascular risk based on the presence and levels of multiple cardiovascular risk factors.

## Current treatment guidelines

With the latest edition of the American College of Cardiology/American Heart Association hypertension treatment guidelines, treatment targets have also become more individualized based on cardiovascular disease risk, with lower blood pressure targets for patients with established CVD or diabetes, as well as in those at a high 10-year predicted cardiovascular risk [3]. Yet, the indications for initiating blood pressure-lowering treatment remain primarily driven by blood pressure levels, whereas for statin treatment, cardiovascular risk instead of cholesterol level is the main driver of recommendations for treatment initiation. In this issue of *PLOS Medicine*, Kazem Rahimi and colleagues, on behalf of the Blood Pressure Lowering Treatment Trialists’ Collaboration, present an analysis of individual patient data indicating that, at the population level, a strategy based on 5-year predicted cardiovascular risk—rather than blood pressure levels alone—could be more effective, especially in a primary prevention setting [4]. For example, they report that compared with a strategy of treating everyone with a systolic blood pressure of 140 mmHg or more, treatment based on overall CVD risk would prevent 3.1% more events (95% CI 1.5% to 5.0%) for the same number of people treated. These data reemphasize that we should target overall cardiovascular risk and aim to modify risk through holistic risk management, as opposed to targeting only individual risk factor levels in isolation.

Traditionally, the focus in cardiovascular risk assessment has been on 10-year probabilities of developing CVD. However, such 10-year absolute cardiovascular risk estimates are often abstract numbers to patients, and risk communication is therefore challenging, especially in younger individuals with multiple risk factors in whom 10-year absolute cardiovascular risk remains low by virtue of their age [5]. For this reason, in the latest iterations of the US and British CVD prevention guidelines [6–8], attention is shifting towards lifetime perspectives. This transition is driven by data demonstrating that cardiovascular risk is best expressed by cumulative exposure to risk factors over time, rather than risk factor levels at a single time point, and aggressive risk factor management should be considered earlier in life [5,6,9,10]. For example, duration of exposure to hypertension in early adulthood is associated with the amount of coronary atherosclerosis in middle age [11]. Barriers towards a full transition from 10-year risk to lifetime risk in preventive cardiology include the lack of established treatment thresholds and suboptimal performance of the available risk models [9].

## Challenges and opportunities

With accruing evidence supporting safe generic medication for blood pressure and cholesterol lowering, indications for treatment have been widened through drastic lowering of treatment thresholds and targets. This is a consistent phenomenon across clinical practice guidelines in high-income countries, to such an extent that nearly all individuals aged 65 years and over now qualify for statin treatment [5,12]. Nonetheless, drug treatment comes with potential side effects and costs. Hence, clinicians and policy makers should retain a healthy level of skepticism towards unrestricted population-wide treatment. On the other hand, broadened indications and widespread use of blood pressure- and cholesterol-lowering medication should not be erroneously labeled as unnecessary medicalization of society [13]: the burden of cardiovascular risk factors remains very high because of unhealthy contemporary lifestyles and justifies medical therapy in a substantial proportion of the population. Therefore, population-wide screening programs for cardiovascular risk assessment could facilitate early detection of those at high risk and identify individuals who would benefit most from early sustained blood pressure- or cholesterol-lowering treatment. However, the optimal age at which to screen for cardiovascular risk factors and subsequent age-specific treatment thresholds and targets are as yet unknown [5,14].

Treatment recommendations from current risk-based prevention guidelines [6–8, 15–17] do not directly reflect the evidence derived from clinical trials [12,18] but rather reflect generalizations of findings derived from clinical trials showing greater absolute risk reduction in those at higher observed cardiovascular risk. Very low-risk individuals may not be recommended preventive treatment although trial evidence of statin efficacy is available for such people [18]. Conversely, findings from trials of antihypertensives and statins are extrapolated to high-risk individuals in whom the efficacy of preventive treatment has not been studied [12,18]. For many of these high-risk individuals, it is highly unlikely that direct trial evidence will ever be accrued, because not every specific patient group can be studied; therefore, extrapolation will always be necessary to some extent. A particularly vulnerable and understudied group consists of persons with comorbidities associated with increased cardiovascular risk, including autoimmune, pulmonary, or liver diseases. Consequently, interactions with drugs used to treat such conditions or disease-specific adverse effects of preventive treatment will remain understudied. Similarly, direct trial evidence on the efficacy of preventive cardiovascular medication in such vulnerable populations will remain absent.

Patient involvement and shared decision-making between patients and physicians has become an increasingly important aspect of CVD prevention. Anticipated cardiovascular risk reduction with blood pressure- or cholesterol-lowering treatment should be weighed against the burden and costs of taking medication, as well as potential adverse effects. In many individuals free of CVD, the risk of developing CVD is relatively low. Hence, patient preferences and attitudes towards preventive medication are key to decisions to initiate or intensify treatment. However, informed decisions rely on a good understanding of an individual’s cardiovascular risk and risk reduction conferred by preventive treatment. Therefore, comprehensible metrics are needed to summarize anticipated treatment benefit, for instance, by expressing lifetime benefits of treatment as gains in CVD-free life expectancy [5,19].

## Future directions

Recent decades have seen a transition from risk factor-based treatment to treatment based on overall short-term and lifetime risk. The work by Rahimi and colleagues in *PLOS Medicine* contributes evidence that risk-based allocation represents a more efficient approach towards blood pressure-lowering treatment in primary prevention of CVD [4]. Now, we should move further towards personalized treatment guidance based on anticipated benefit [5,19,20]. Anticipated treatment benefit is related to absolute overall risk of CVD but is not simply a rescaling of risk.

First, data from clinical trials on pharmacological interventions need to be incorporated in the construction of estimates of anticipated treatment benefit [20–22]. For instance, blood pressure- and cholesterol-lowering treatment remains effective at older age, yet the relative risk reduction generally diminishes with age. Similarly, treatment effects may vary by ethnicity, by specific risk factor levels, and by comorbidity, such as renal disease. For example, let us compare 2 healthy men of similar age with identical 10-year cardiovascular risk estimates. One man’s risk is driven by moderate hypertension, and the other man’s risk by moderate hypercholesterolemia: it seems most reasonable to lower cardiovascular risk with antihypertensives in the former and with statins in the latter [20]. Second, since preventive treatment to lower blood pressure and cholesterol is often initiated with the intention of being used for decades or even lifelong, life expectancy is a key variable in estimating anticipated benefit. For instance, smoking roughly doubles the risk of CVD, yet anticipated gains in CVD-free life expectancy with lifelong statin therapy are similar for smokers and nonsmokers. This is caused by competing risks of smoking-related cancers and other life-limiting diseases [19].

Combining information on demographics, risk factor levels, and data on relative risk reductions obtained from clinical trials can inform models to provide anticipated benefits expressed as gains in CVD-free life expectancy with specific treatment options and thereby facilitate more informed treatment decisions. Similar estimates of anticipated harms can be produced for the risks associated with drug treatment. Healthcare policy makers can use such data to project costs and benefits of treatment and thereby provide clinicians with generally acceptable treatment thresholds.

CVD remains a leading cause of morbidity and mortality worldwide. Despite great progress in treatment of acute cardiovascular conditions, first manifestations of CVD are still often lethal or result in long-term disability. Therefore, optimal primary prevention should remain a priority in the future. More new strategies will emerge, and clinical practice guidelines are likely to evolve from risk factor-based treatment to risk-based treatment and beyond to treatment based on anticipated benefit. Throughout this process, cardiovascular specialists, general practitioners, and researchers need to keep prioritizing time and resources to offer preventive measures to healthy individuals free from CVD and focus on compliance and persistence with treatment, irrespective of the prevention strategy chosen.

## Abbreviations

CVD: cardiovascular disease
